# Supplementary material for: Real-Time Tracheal Ultrasound vs. Capnography for Intubation Confirmation during CPR Wearing a Powered Air-Purifying Respirator in COVID-19 Era
Source: Diagnostics (Basel). 2024 Jan 21;14(2):225. doi: 10.3390/diagnostics14020225 (PMC10813934; doi:10.3390/diagnostics14020225)
Supplement: Supplementary file 1 [file diagnostics-14-00225-s001.zip › SUPPLEMENTAL DATA 230116.pdf]

SUPPLEMENTARY DATA

Supplementary Table S1. Relation between TTUS proficiency and image acquisition time

Experience in TTUS	Time spent on TTUS (s) (Median [IQR])	Kruskal–Wallis test	Jonckheere–Terpstra trend test
0–1 (n = 1)	2 [2–2]	*P*-value = 0.3072	*P*-value = 0.1446
2–5 (n = 5)	10 [8–6]		
6–10 (n = 5)	10 [10–20]		
> 10 (n = 22)	15 [9–25]		

The number of TTUS experiences and time required for TTUS tube placement confirmation using the Kruskal–Wallis test not shows a significant correlation. The Jonckheere–Terpstra trend test demonstrates no trend between TTUS proficiency and time spent on TTUS.
TTUS, trans-tracheal ultrasound; IQR, interquartile range

Supplementary Video S1 Endotracheal intubation at TTUS scan

TTUS, trans-tracheal ultrasound

Supplementary Video S2 Esophageal intubation at TTUS scan

TTUS, trans-tracheal ultrasound

Supplementary Video S3 Lung sliding assessment
